# Correction: Schmitz et al. Validation of the Palliative Care and Rapid Emergency Screening (P-CaRES) Tool in Germany. *J. Clin. Med.* 2025, *14*, 2191

**DOI:** 10.3390/jcm15051720

**Published:** 2026-02-25

**Authors:** Julia Schmitz, Mitra Tewes, Baicy Mathew, Marie Bubel, Clemens Kill, Joachim Risse, Eva-Maria Huessler, Bernd Kowall, Maria Rosa Salvador Comino

**Affiliations:** 1 Department of Palliative Medicine, University Hospital Essen, University of Duisburg-Essen, Hufelandstr. 55, 45147 Essen, Germany; julia.schmitz@ext.uk-essen.de (J.S.); mitra.tewes@uk-essen.de (M.T.); baicy.mathew@uk-essen.de (B.M.);; 2 Center of Emergency Medicine, University Hospital Essen, Hufelandstr. 55, 45147 Essen, Germany; clemens.kill@uk-essen.de (C.K.);; 3 Institute for Medical Informatics, Biometry and Epidemiology at the University Hospital Essen, Hufelandstr. 55, 45122 Essen, Germanybernd.kowall@uk-essen.de (B.K.)

## Error in Figure

In the original publication [1], there was a mistake in Figure 2 as published. The validated version of the P-CaRES tool included in the article is missing one item in the first section of the tool. Figure 2 currently shows only seven items in Part 1 of the P-CaRES tool. The corrected figure should include eight items, according to the final validated version of the tool.

The missing item is “Subjektive Einschätzung des Befragers—Erhöhtes Risiko eines vorzeitigen Versterbens: Beispiele: Hüftfraktur > Alter von 80; schwerwiegende Traumata bei Senioren (multiple Rippenfrakturen, intrakranielle Blutungen), fortgeschrittenes AIDS, etc.” This corresponds to the English item “Provider Discretion—High chance of Accelerated Death. Examples: Hip fracture, Major trauma in the elderly, Advanced AIDS, etc.”, as shown in Figure 1, which displays the original tool in English.

The corrected Figure 2 appears below. The authors state that the scientific conclusions are unaffected. This correction was approved by the Academic Editor. The original publication has also been updated.

## Figures and Tables

**Figure 2:**
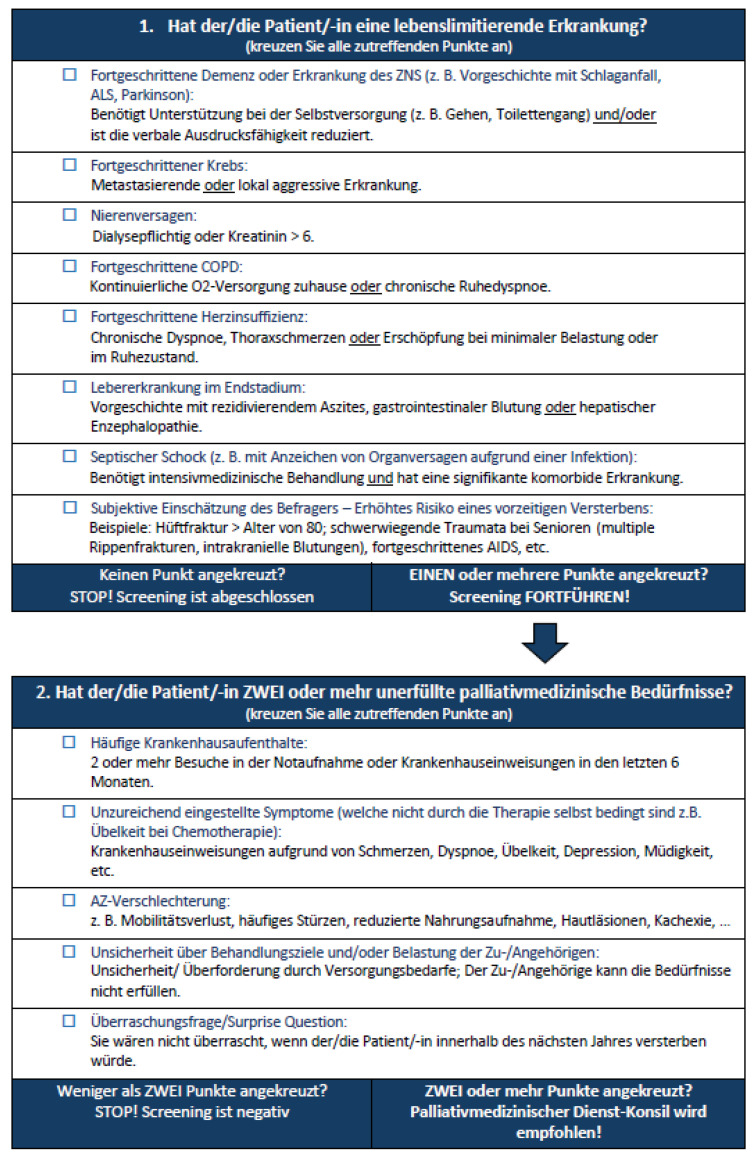
German translated and validated version of the P-CaRES tool.
