# Supplementary material for: Validation of the Palliative Care and Rapid Emergency Screening (P-CaRES) Tool in Germany
Source: J Clin Med. 2025 Mar 23;14(7):2191. doi: 10.3390/jcm14072191 (PMC11989907; doi:10.3390/jcm14072191)
Supplement: Supplementary file 1 [file jcm-14-02191-s001.zip › jcm-3512769-supplementary.pdf]

Supplemental Material

Supplemental Figure S1. SPICT tool (original English version)

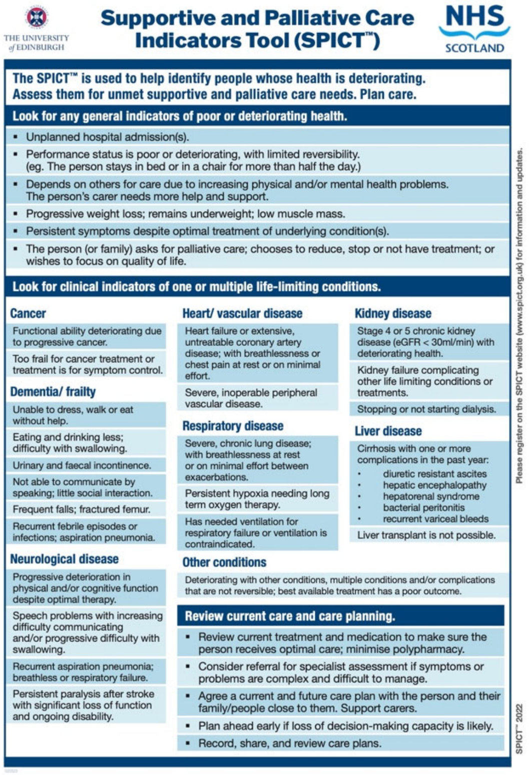

THE UNIVERSITY of EDINBURGH

Supportive and Palliative Care Indicators Tool (SPICT™)

NHS SCOTLAND

The SPICT™ is used to help identify people whose health is deteriorating. Assess them for unmet supportive and palliative care needs. Plan care.

Look for any general indicators of poor or deteriorating health.

- Unplanned hospital admission(s).
- Performance status is poor or deteriorating, with limited reversibility. (eg. The person stays in bed or in a chair for more than half the day.)
- Depends on others for care due to increasing physical and/or mental health problems. The person's carer needs more help and support.
- Progressive weight loss; remains underweight; low muscle mass.
- Persistent symptoms despite optimal treatment of underlying condition(s).
- The person (or family) asks for palliative care; chooses to reduce, stop or not have treatment; or wishes to focus on quality of life.

Look for clinical indicators of one or multiple life-limiting conditions.

Cancer

Functional ability deteriorating due to progressive cancer.

Too frail for cancer treatment or treatment is for symptom control.

Dementia/ frailty

Unable to dress, walk or eat without help.

Eating and drinking less; difficulty with swallowing.

Urinary and faecal incontinence.

Not able to communicate by speaking; little social interaction.

Frequent falls; fractured femur.

Recurrent febrile episodes or infections; aspiration pneumonia.

Neurological disease

Progressive deterioration in physical and/or cognitive function despite optimal therapy.

Speech problems with increasing difficulty communicating and/or progressive difficulty with swallowing.

Recurrent aspiration pneumonia; breathless or respiratory failure.

Persistent paralysis after stroke with significant loss of function and ongoing disability.

Heart/ vascular disease

Heart failure or extensive, untreatable coronary artery disease; with breathlessness or chest pain at rest or on minimal effort.

Severe, inoperable peripheral vascular disease.

Respiratory disease

Severe, chronic lung disease; with breathlessness at rest or on minimal effort between exacerbations.

Persistent hypoxia needing long term oxygen therapy.

Has needed ventilation for respiratory failure or ventilation is contraindicated.

Kidney disease

Stage 4 or 5 chronic kidney disease (eGFR < 30ml/min) with deteriorating health.

Kidney failure complicating other life limiting conditions or treatments.

Stopping or not starting dialysis.

Liver disease

Cirrhosis with one or more complications in the past year:

- diuretic resistant ascites
- hepatic encephalopathy
- hepatorenal syndrome
- bacterial peritonitis
- recurrent variceal bleeds

Liver transplant is not possible.

Other conditions

Deteriorating with other conditions, multiple conditions and/or complications that are not reversible; best available treatment has a poor outcome.

Review current care and care planning.

- Review current treatment and medication to make sure the person receives optimal care; minimise polypharmacy.
- Consider referral for specialist assessment if symptoms or problems are complex and difficult to manage.
- Agree a current and future care plan with the person and their family/people close to them. Support carers.
- Plan ahead early if loss of decision-making capacity is likely.
- Record, share, and review care plans.

Please register on the SPICT website (www.spict.org.uk) for information and updates.

SPICT™ 2022

Supplemental Figure S2. Validated German SPICT too

THE UNIVERSITY of EDINBURGH

Supportive and Palliative Care Indicators Tool (SPICT-DE™)

SPICT-DE™ ist ein Leitfaden zur Identifikation von Patienten, die von einer Palliativversorgung profitieren können, und bei denen ein palliatives Basisassessment sowie eine palliative Versorgungsplanung angezeigt sind.

Allgemeine Indikatoren, die auf eine Verschlechterung des Gesundheitszustandes hindeuten können:

- (Mehrfache) ungeplante Krankenhauseinweisungen.
- Reduzierter Allgemeinzustand oder zunehmende Verschlechterung; Verbesserung ist unwahrscheinlich (z.B. Patient verbringt mehr als den halben Tag liegend oder sitzend).
- Patient ist aufgrund körperlicher und/oder seelischer Beeinträchtigungen im Alltag auf Unterstützung angewiesen.
- Pflegende Angehörige benötigen (zusätzliche) Unterstützung/Entlastung.
- Progredienter Gewichtsverlust; persistierendes Untergewicht; geringe Muskelmasse.
- Anhaltende belastende Symptome trotz optimaler Therapie der zugrunde liegenden Erkrankung(en).
- Patient/Angehörige signalisieren den Wunsch nach Palliativversorgung, Therapiebegrenzung/ Therapiezieländerung.

Spezifische Indikatoren, wenn Erkrankungen im fortgeschrittenen Stadium vorliegen:

Krebserkrankung

Progredienz mit zunehmender Symptomlast und funktionalen Einschränkungen.

Patient ist zu schwach für eine Tumortherapie bzw. primäres Ziel ist die Symptomkontrolle.

Demenz/Gebrechlichkeit

Multiple Beeinträchtigungen, z.B.:

Hilfe beim Anziehen, Gehen oder Essen erforderlich.

Essen und Trinken vermindert; zunehmende Schluckstörungen.

Harn- und Stuhlinkontinenz.

Verbale Kommunikation und/oder soziale Interaktion kaum möglich.

Wiederholte Sturzereignisse; Femurfraktur.

Wiederholte fieberhafte Episoden bzw. Infekte; Aspirationspneumonie(n).

Neurologische Erkrankung

Progredienz mit zunehmenden körperlichen und/oder kognitiven Einschränkungen trotz optimaler Therapie, z.B.:

Zunehmende Sprachstörungen und eingeschränkte soziale Interaktion.

Zunehmende Schluckstörungen, Aspirationspneumonien; Atemnot und/oder Lungenversagen.

Anhaltende Lähmungen nach Schlaganfall mit eingeschränkter Funktionsfähigkeit/Behinderung.

Kardiovaskuläre Erkrankung

Herzinsuffizienz oder ausgeprägte koronare Herzerkrankung mit Atemnot oder Thoraxschmerz in Ruhe oder bei geringer Belastung.

Schwere, inoperable periphere Gefäßerkrankung.

Atemwegserkrankung

Fortgeschrittene chronische Lungenerkrankung mit Atemnot in Ruhe/bei geringer körperlicher Belastung.

Benötigt Sauerstofftherapie aufgrund anhaltender Hypoxämie.

Zustand nach Lungenversagen; (erneute) Beatmung ist nicht erfolgversprechend.

Nierenerkrankung

Chronische Niereninsuffizienz mit deutlicher AZ-Verschlechterung.

Niereninsuffizienz als komplizierender Faktor anderer Erkrankungen/Behandlungen.

Beendigung oder Verzicht auf Einleitung einer Dialyse wird erwogen.

Lebererkrankung

Leberzirrhose mit Komplikationen in den letzten 12 Monaten, z.B.:

- Diuretikum-resistenter Aszites
- hepatische Enzephalopathie
- hepatorenales Syndrom
- bakterielle Peritonitis
- rezidivierende Ösophagusvarizenblutungen

Lebertransplantation nicht angezeigt bzw. nicht erfolgversprechend.

Andere lebenslimitierende Erkrankungen

AZ-Verschlechterung aufgrund anderer nicht reversibler Gesundheitsprobleme, bei denen eine kurative Therapie wenig erfolgversprechend ist.

Mögliche Maßnahmen und nächste Schritte:

- Überprüfung der medikamentösen/nicht-medikamentösen Therapie zur Versorgungsoptimierung und Vermeidung von Polypharmazie.
- Überprüfung, ob ein palliatives Konsil/eine spezialisierte Palliativversorgung angezeigt ist, z.B. bei schwer kontrollierbaren Symptomen, komplexen Versorgungsproblemen.
- Klärung der Therapieziele/Versorgungsplanung mit dem Patienten und der Familie.
- Klärung von Unterstützungsbedarf/Entlastungsangeboten für pflegende Angehörige.
- Frühzeitige Einleitung einer vorausschauenden Versorgungsplanung bei erhöhtem Risiko des Verlusts der Entscheidungskompetenz.
- Dokumentation, Kommunikation und Koordination des Versorgungsplans.

SPICT-DE™ APRIL 2019 Translation and adaption by Hannover Medical School Bitte melden Sie sich auf der SPICT Website für Informationen und Updates an (www.spict.org.uk)

Supplemental Table S1

	Positive Items				Statistic values	
	P-CaRES		SPICT		Kappa	95%-CI
	n	%	n	%		
**Test Items**						
CNS Disease/ Advanced Dementia	13	5.0	17	6.6	0.65	[0.4, 0.9]
Frailty	--	--	38	14.7	--	--
Advanced Cancer	33	12.8	36	14.0	0.95	[0.9, 1]
End Stage Renal Disease	1	0.4	2	0.8	0.66	[0, 1]
Advanced COPD/ Lung disease	9	3.5	9	3.5	0.88	[0.7, 1]
Advanced Heart Disease	8	3.1	7	2.7	0.79	[0.6, 1]
End Stage Liver Disease	2	0.8	2	0.8	1	[1]
Septic Shock	0	0.0	--	--	--	--
Provides Discretion	4	1.6	--	--	--	--
Frequent visits	44	65.7 (17.1*)	113	43.8	0.9	[0.8, 1]
Uncontrolled Symptoms	40	59.7 (15.5*)	78	30.2	0.73	[0.6, 0.9]
Functional Decline	34	50.7 (13.2*)	49	19.0	0.91	[0.8, 1]
Uncertainly about Goals-of-Care	0	0.0	--	--	--	--
Caregiver Distress	2	3.0 (0.8*)	--	--	--	--
Wish for palliative therapy	--	--	3	1.2	--	--
Weight loss/ underweight/ low muscle mass	--	--	55	21.3	--	--
Everyday support/ care by relatives	--	--	79	30.6	--	--
Surprise Question	23	34.3 (8.9*)	--	--	--	--
Screening positive	50	19.4	68	26.4	0.69	[0.6, 0.8]

CI: confidence interval, CNS: central nervous system, COPD: chronic obstructive pulmonary disease
* Percentage of all screened patients, including those who did not have a positive item in the first part of the screening

Supplemental Table S1. Comparison of the SPICT and P-CaRES tools

To determine construct validity, we screened 258 emergency department patients using both tools and compared their results.
